# Macrophage Autophagy and Silicosis: Current Perspective and Latest Insights

**DOI:** 10.3390/ijms22010453

**Published:** 2021-01-05

**Authors:** Shiyi Tan, Shi Chen

**Affiliations:** Key Laboratory of Molecular Epidemiology of Hunan Province, Hunan Normal University, Changsha 410013, China; eleven@smail.hunnu.edu.cn

**Keywords:** alveolar macrophage, autophagy, silicosis

## Abstract

Silicosis is an urgent public health problem in many countries. Alveolar macrophage (AM) plays an important role in silicosis progression. Autophagy is a balanced mechanism for regulating the cycle of synthesis and degradation of cellular components. Our previous study has shown that silica engulfment results in lysosomal rupture, which may lead to the accumulation of autophagosomes in AMs of human silicosis. The excessive accumulation of autophagosomes may lead to apoptosis in AMs. Herein, we addressed some assumptions concerning the complex function of autophagy-related proteins on the silicosis pathogenesis. We also recapped the molecular mechanism of several critical proteins targeting macrophage autophagy in the process of silicosis fibrosis. Furthermore, we summarized several exogenous chemicals that may cause an aggravation or alleviation for silica-induced pulmonary fibrosis by regulating AM autophagy. For example, lipopolysaccharides or nicotine may have a detrimental effect combined together with silica dust via exacerbating the blockade of AM autophagic degradation. Simultaneously, some natural product ingredients such as atractylenolide III, dioscin, or trehalose may be the potential AM autophagy regulators, protecting against silicosis fibrosis. In conclusion, the deeper molecular mechanism of these autophagy targets should be explored in order to provide feasible clues for silicosis therapy in the clinical setting.

## 1. Silica Dust and Silicosis

Silicosis is characterized by silicon nodules and diffuse pulmonary fibrosis caused by long-term inhalation of crystalline free silica (SiO_2_) dust in the workplaces [1]. In the following discussion, the silica we mention refers to crystalline silica. Silicosis fibrosis will lead to severe lung function damage, whose degree presents a trend of progressive increase. Silicosis patients often have different respiratory symptoms such as cough, sputum expectoration, shortness of breath, chest tightness, and discomfort. As silicosis worsens, it presents different complications, including tuberculosis, respiratory tract infection, respiratory insufficiency, and even pneumothorax [2]. Silicosis is a serious public health problem which threatens workers’ physical and mental health. At present, the silicosis pathogenesis has not been fully elucidated.

### 1.1. Influencing Factors of Silica Dust to Lung Toxicity and Related Derivative Drugs

Normally, silica can be inhaled when producing or utilizing industrial raw materials such as sand, gravel, industrial minerals, coal, cement, and plaster, etc. A recent study shows that the silanol group on the surface of silica may be the crucial active part resulting in silicosis fibrosis [3]. Simultaneously, other properties of silica like crystallinity and fracturing are identified as influencing factors of its toxicity. In addition to the intrinsic decisive factors, the silica’s surface state also contributes to silica toxicity [4]. In response to such a mechanism, polyvinyl-pyridine-N-oxide (PVNO) has been applied in the treatment of silicosis patients in clinical trials. PVNO forms a polymer film on the surface of silica and further changes the silicic acid’s hydrogen transfer effect. Silica treated by PVNO reduces the disruption of lysosomes relatively, protecting alveolar macrophage (AM) against the toxic effects of silica [5,6,7]. Additionally, broad-spectrum anti-fibrosis drug pirfenidone, currently undergoing clinical trials, has a mechanism aimed at reducing the proliferation and differentiation of fibroblasts [8,9]. Currently, there are no drugs targeting the cause of silicosis, i.e., silica invasion. As a complex “stimulus”, silica cannot be eliminated from the lungs by the drugs mentioned above. Considering there are no effective drugs to reverse silica-induced pulmonary fibrosis, it is important to explore complex mechanisms and therapeutic targets of silicosis.

### 1.2. Silicosis Lesion Changes and Animal Model Establishments

Microscopically early lesions present the appearance of cellular nodules, which are composed of the focal accumulation of dust cells. Macrophages are referred to as dust cells after swallowing silica. With time, typical silicon nodules, developed from cellular nodules, appear as distinctly concentric, arranged around the collagenous central region [10]. Common methods of the silicosis animal model establishment include intratracheal instillation and inhalation (Table 1), of which one-time non-exposed intratracheal instillation is most commonly used. According to the changes in silica-induced lesions, after instilling SiO_2_ suspension into the trachea, the observation of inflammation can be selected on the 7th day. Normally, typical silicon nodules can be observed after the 28th day, usually 7 days as a unit, that is, 42 days, 56 days, or longer [11,12,13]. However, whether it is SiO_2_ aerosol or SiO_2_ suspension, both have to have been sterilized before modeling. Compared to the actual inhalation condition of silicosis patients, the silicosis animal models cannot evaluate the joint toxic effect of the silica together with any minerals, metals, or bacterial matter on its surface. Therefore, in future studies, the combined effect of silica and other poisons, not silica only, should be considered.

## 2. Alveolar Macrophage, a “Gatekeeper” for Defense against Silica Dust

In clinical trial, only whole-lung lavage (WLL) can clear silica from the patients’ alveoli cavity, even a small part from the lung interstitium, improving the respiratory function of silicosis patients [17,18]. Bronchoalveolar lavage fluid (BALF) collected by WLL contains more than 90% AMs. AM is in the alveolar surfactant film and the only macrophage exposed to air [19,20]. As a “gatekeeper”, AM phagocytosis is necessary for protecting against silica invasion [21,22]. Therefore, the condition of AM essentially determines the degree of recovery of the lung.

Once it enters into AMs through a class A scavenger receptor [23], silica will be engulfed by AMs and then degraded by their lysosome. In this process of phagocytosis, the lysosomal membrane is damaged by the H-bonding reaction, which allows enzymes to leak into the cytoplasm continuously and excessively, leading to disintegration, necrosis, or apoptosis of AMs [24,25]. Among these, AM apoptosis is a kind of paramount pathogenesis involved in silica-induced pulmonary fibrosis. Apoptotic AMs secrete a large number of inflammatory factors and further cause an inflammation cascade. Subsequently, the proliferation, activation, and migration of pulmonary fibroblasts synthesize and secrete collagen, resulting in eventual silicosis fibrosis [26,27]. Additionally, during the process of silicosis fibrosis, the level of AM apoptosis will escalate gradually due to failed phagocytosis or abnormalities in the clearance of the apoptotic AMs, aggravating such a cascade reaction [28]. Overall, the apoptotic response occurring at an early stage of the silicosis progression has a compensatory function to remove injured cells and clear inflammation, resulting in lung tissue remodeling. However, subsequent aggravation of silicosis occurs as AM apoptosis increases continuously.

**Figure 1:**
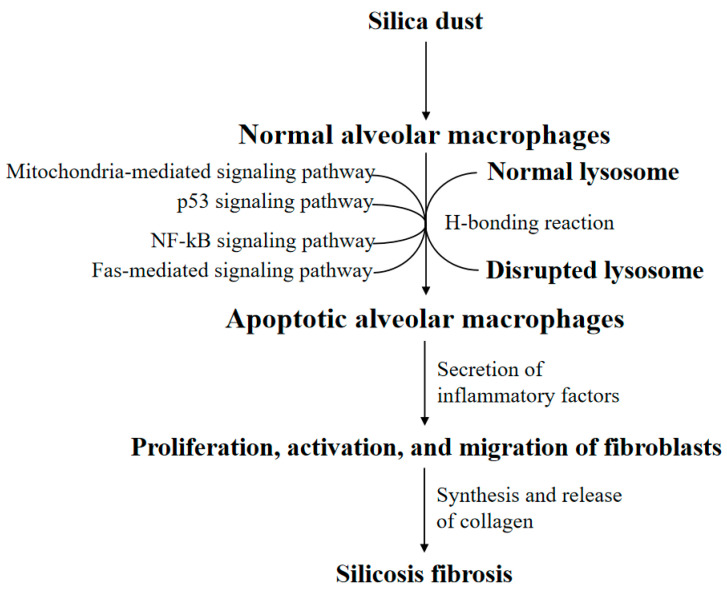
The general silicosis pathological mechanism. The attack of silica dust triggers the phagocytosis of alveolar macrophages (AMs). However, the lysosomal membrane of AM will be disrupted by the H-bonding reaction, causing AM apoptosis. Notably, AM apoptosis may be regulated by the mitochondria apoptotic pathway, Fas apoptotic pathway, nuclear factor kappa-B (NF-κB) apoptotic pathway, and p53 apoptotic pathway. Apoptotic AM secrets a series of inflammatory factors. Eventually, the proliferation, activation, and migration of fibroblasts synthesize and release collagen, resulting in silicosis fibrosis.

Many types of research have demonstrated that the silica-induced AM apoptosis follows the intrinsic cell apoptosis program mediated by the mitochondria [29,30]. Also, silica aggravates AM apoptosis through a Fas-mediated exogenous pathway [31,32]. Simultaneously, silica-induced RAW 264.7 cell apoptosis aggravates through the inhibition of nuclear factor kappa-B (NF-κB) signaling pathways. In contrast with RAW 264.7 cells, inhibition of NF-κB activity in IC-21 cells prevents silica-induced apoptosis [33]. Different macrophage cell lines present a converse cell apoptosis level when inhibiting the nuclear transfer of NF-κB, suggesting the relationship between NF-kB and pulmonary macrophage apoptosis deserves further exploration in relation to the development of silicosis. Silica also facilitates apoptosis through the p53 signaling pathway [34]. The general pathological mechanism of silicosis has been shown in Figure 1. Macrophage apoptosis, especially AM apoptosis, has been regarded as one of the effect indicators of silicosis progression in many studies. Meanwhile, the regulation for these apoptotic signaling pathways above may be a potential intervention for silicosis therapy.

## 3. Some Views about the Autophagy-Related Proteins and Silicosis Pathogenesis

Autophagy is responsible for maintaining cell homeostasis through a balanced regulation between the synthesis and degradation of intracellular components. It occurs merely in eukaryotic cells. Four steps exist in autophagy: (1) Initiation of autophagy; (2) Formation of autophagosome; (3) Fusion of autophagosome and lysosome; (4) Degradation by autophagolysosome [35]. It is worth mentioning that there is extensive cross-talk between autophagy and innate immunity, including the influence of autophagy on macrophage’s phagocytosis, the release of inflammatory cytokines, antigen presentation, and expression of different membrane receptors [36]. The immune regulatory effects mediated by autophagy need to be further elucidated. Furthermore, the entire process of autophagy is regulated by different autophagy-related proteins (ATG) at all times. They are all essential molecules for autophagy and participate in different stages of autophagy. Here, we propose some new views concerning the complex role of these ATG in the process of silicosis.

### 3.1. Static Detection of LC3-II Alone Is Not Sufficient to Reflect Autophagy Activity

Many ATGs can assess the autophagy activity, such as unc-51 like kinase 1 (ULK1), microtubule-associated protein 1A/1B-light chain 3 (LC3), sequestosome 1 (p62/SQSTM1), lysosome-associated membrane protein (LAMP), Beclin1, etc. Different autophagy markers aim at different links of the autophagy process. For example, p62 has been regarded as a marker of the process of autophagic degradation due to coupling with ubiquitin-tagged misfolded proteins. LAMP is used to detect whether lysosomal structure is complete or not. Others like ULK1 and Beclin1 can detect the change of autophagy activity through monitoring autophagy initialization [37]. Among them, the ratio of LC3-II/LC3-I is the most commonly used. LC3-I ubiquitination couples phosphatidylethanolamine to generate lipidated LC3-II, which is recruited in the surface of the autophagosome. LC3-II, together with the wrapped contents within the autophagosome, will be degraded by the lysosome [38]. The ratio of LC3-II/I refers to the turnover of LC3-II from synthesis to degradation. As a marker of the autophagosome, the increased or decreased ratio of LC3-II/I is always considered in the induction or inhibition of autophagy activity [39]. However, such a view is not exactly correct. The raised LC3-II/I ratio indicates enhanced synthesis of autophagosomes, resulting from the accumulation of autophagosomes, the enhancement of autophagy activity, or the blockade of autophagic degradation [40,41]. Therefore, the simple detection of LC3 by immunoblotting or immunofluorescence cannot reflect autophagy activity. Currently, many studies utilize various methods to monitor autophagy flux. (1) In dynamic detection, like the dual fluorescence mRFP-GFP-LC3 system, static technology merely detects the outcome of autophagy. However, dynamic technology focuses on whether the process of autophagy is unobstructed or not. (2) Combination of p62 and LC3-II can further determine whether the degradation function of autophagy impairs or not. (3) It can be judged which link of the process of autophagy goes wrong by the comparison of LC3-II levels in the absence and presence of the autophagy inhibitor of different molecular mechanisms.

### 3.2. p62 May Perform a Complex Function in the Development of Silicosis

During the process of autophagy, the ubiquitin-tagged misfolded proteins, coupled with p62, are assembled into aggregates, then are engulfed and degraded [42]. Therefore, the accumulation of p62 means the blockade of autophagic degradation. Notably, p62 is a negative regulator response for Toll-like receptor 4 (TLR4) activation. The latter is an important pattern recognition receptor (PRR), which is responsible for binding the pathogen-associated molecular patterns (PAMPs) to act as a defense mechanism against invading pathogens. Normally, tumor necrosis factor receptor-associated factor 6 (TRAF6)-Beclin1 complex forms during the engagement of TLR4 and its ligand. TRAF6 thereby induces the ubiquitination of Beclin1, activating autophagy activity. However, p62 can competitively inhibit the interaction of Beclin1 and TRAF6, attenuating TRAF6-induced Beclin1 ubiquitination [43]. It can be further assumed that autophagy dysfunction regulated by the interaction of p62 and TRAF6-Beclin1 complex is involved in the pathogenesis of silicosis.

Positive feedback between p62 and NF-E2-related factor 2 (Nrf2) has been attracted much attention. When autophagy is impaired, accumulated p62 sequesters Kelch-like ECH-associated protein 1 (Keap1) into aggregates. Nrf2 is subsequently separated from Keap1, leading to the activation of the antioxidant response element. Meanwhile, the p62 gene is an Nrf2 target, Nrf2 thereby induces the production of p62 [44]. Nrf2 should have performed an anti-oxidant function. However, Nrf2 is activated excessively in the Atg7- and p62-deficient mice, leading to liver injury. Furthermore, there is no liver injury in Nrf2-deficient mice [45]. It suggests that p62 accumulation caused by the impairment of autophagy may enhance the activation of Nrf2 until pathological damage occurs. The reason may be that the combination of p62 and Keap1 leads to a lack of degradation mechanism of Nrf2 [46]. Notably, the characteristic of the chemoresistance of Nrf2 has also been attracting much attention. Recent research has demonstrated that KRAS interferes with the metabolism of glutamine through upregulating Nrf2, bolstering the effect of gemcitabine against pancreatic cancer [47]. Collectively, the findings above raise the possibility that p62 accumulation induced by the blockade of autophagic degradation may lead to the disruption of Nrf2 anti-oxidant function in the development of silicosis. Moreover, Nrf2 chemoresistance implies that more characteristics of Nrf2 should be explored. The detailed understanding of the Nrf2 function or interaction between Nrf2 and p62-mediated autophagy may facilitate the discovery of new therapies for silicosis.

## 4. Macrophage Autophagy Plays an Important Role in the Silicosis Progression

Silica dust particles have been observed in AM autophagolysosome of silicosis patients. Moreover, abnormal autophagy activity has been observed in the lung tissues of the silicosis rat model [48,49]. These findings suggest that AM autophagy is associated with silicosis. Another study has shown that with the development of human silicosis, AM lysosomes gradually decrease, whereas AM autophagosomes increase; furthermore, the expression of BCL2-Associated X (Bax) and cleaved caspase3 increase, whereas the expression of BCL2 decreases [50]. Therefore, the blockade of autophagic degradation may aggravate AM apoptosis in human silicosis.

### 4.1. Some Critical Proteins Targeting Autophagy Exist in Silicosis Pathogenesis

**Figure 2:**
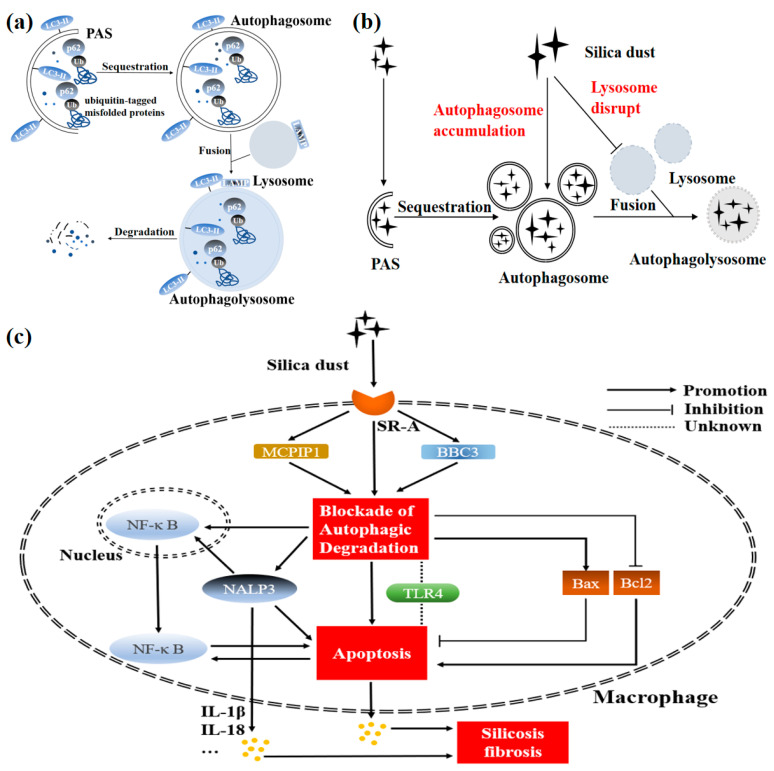
Determined mechanism of silicosis pathogenesis mediated by macrophage autophagy. (**a**) Normally, the pre-autophagosomal structure (PAS) begins to engulf cytosolic components, following which the signal of autophagy is activated. Subsequently, the autophagosomal membrane expands, matures, and closes gradually. Once closed, the autophagosome will fuse with the lysosome to form the autophagolysosome, degrading its wrapped contents. Several autophagy-related proteins act critically, indicating effect during the process of autophagy. Sequestosome 1 (p62/SQSTM1) interacts with ubiquitinated substrates, participating in the subsequent process of autophagic degradation. Microtubule-associated protein 1A/1B-light chain 3-I (LC3-I) conjugates with phosphatidylethanolamine to form LC3-II, involved in the expansion, maturation, and closure of autophagosome. Additionally, lysosome-associated membrane protein (LAMP) is attached to the surface of the lysosome. (**b**) When invading macrophages (especially alveolar macrophages) excessively, silica dust will cause autophagosomes to accumulate and lysosomes to disrupt (i.e., the dysfunction of the autophagy-lysosomal system). (**c**) Silica dust invades macrophages (especially alveolar macrophages) via a class A scavenger receptor (SR-A) and causes the blockade of autophagic degradation. The impairment of autophagy function caused by silica further leads to excessive macrophage apoptosis by decreasing the level of BCL2 and increasing the level of BCL2-Associated X (Bax). The inflammation and subsequent silicosis fibrosis occur eventually. Notably, nuclear transfer of nuclear factor kappa-B (NF-κB) may be an important link between autophagy and apoptosis in silicosis. In this pathological progression, disruption of the autophagy-lysosomal system induces macrophage apoptosis through the activation of NACHT-, LRR-, and PYD domain-containing protein 3 (NALP3). NALP3 also increases macrophage apoptosis via the nuclear transfer of NF-κB. Meanwhile, activation of BCL2-binding component 3 (BBC3) or Monocyte chemoattractant protein-1-induced protein 1 (MCPIP1) promotes macrophage apoptosis through exacerbating the autophagic degradation. However, the functional role of Toll-like receptor 4 (TLR4) is still controversial.

Some critical proteins have been recognized as effective targets regulating autophagy in silicosis progression (Figure 2). Many studies have shown the increased number of the typical autophagosomal double membrane and enhanced conversion of LC3-I to LC3-II in macrophages treated with a TLR4 ligand lipopolysaccharide (LPS) [51,52]. TLR4 thereby is viewed as a positive regulator of autophagy in macrophages. Currently, the functional role of TLR4 in silica-induced pulmonary fibrosis is controversial. The level of TLR4 mRNA is upregulated with the time interval increases in the U937-differentiated macrophages after silica treatment [53]. However, with the development of human silicosis, the expression of TLR4 is reduced in AMs [50]. Compared to the U937-differentiated macrophages with one-time exposure to silica, AMs from lung tissues of silicosis patients may reflect workers’ actual silica absorption better. Furthermore, TLR4 inhibitor TAK-242 suppresses the release of Interleukin-1β (IL-1β) and IL-6 in the U937-differentiated macrophages exposed to silica, suggesting the activation of TLR4 may intensify silica-induced silicosis fibrosis [53]. The adverse effect of TLR4 activation has also been confirmed in bleomycin-induced, LPS-induced pulmonary fibrosis models [54,55]. Oppositely, another study suggests knockout of TLR4 may aggravate silica-induced pulmonary fibrosis [56]. TLR4 is a critical targeted protein not only in PRR-PAMPs recognition but in autophagy induction. TLR4, whose complete silence or excessive activation both produce damage to the body, needs to possibly be maintained at a normal level. How does the TLR4 activity changes with the development of silicosis? What is its specific mechanism? Are treatments targeting autophagy activity regulated by TLR4 expected to be an effective therapy for intervention in silicosis? Or is there cross-talk between the TLR4-regulated function of the immune system and the autophagy process? These questions deserve further investigation.

Other apoptosis or inflammation-related proteins can also regulate autophagy in silica-induced pulmonary fibrosis. BCL2-binding component 3 (BBC3), which belongs to the BCL2 family, is a potent activator of apoptosis. BBC3 can inhibit competitively the binding of Beclin1 and Bcl2, thereby facilitating autophagy activity [57]. Through the comparison of autophagy levels in BBC^−/−^ or BBC^+/+^ U937-differentiated macrophages treated with silica, researchers found that autophagy may be mediated by BBC3. Moreover, BBC3-mediated autophagy facilitates apoptosis and the subsequent pro-fibrotic effect by silica on pulmonary tissues [58]. Thus, the knockdown of BBC may be the targeting point for anti-fibrosis in silicosis development. Moreover, Monocyte chemoattractant protein-1 (MCP-1), an inflammatory mediator secreted by activated AMs, can regulate silica-induced pulmonary fibroblast migration [59,60]. MCP-1-induced protein 1 (MCPIP1) is a pivotal downstream molecule of MCP-1. After silica treatment, MCPIP1 activates autophagy activity, further aggravating the silicosis progression through the p53 signaling pathway in the U937-differentiated macrophages. Meanwhile, MCPIP1-siRNA reverses silica-induced autophagy and apoptosis of U937-differentiated macrophages [61,62]. These results demonstrate that the inhibition of MCPIP1 may serve as a promising therapeutic strategy to prevent silicosis.

Cytoplasmic receptor NACHT-, LRR-, and PYD domain-containing protein 3 (NALP3) can be activated by silica in AMs of silicosis [63]. NALP3 activation not only drives the release of caspase-1, IL-1β, and IL-18 but stimulates the nuclear transfer of NF-κB, thereby boosting AM apoptosis and inflammation [64]. Notably, NALP3 can be activated by silica-induced lysosomal swelling and damage [65,66], suggesting that the important role of NALP3 in AM apoptosis and inflammation of silicosis may correlate with the changes of autophagy-lysosomal system function. Recent studies have linked autophagy and NALP3 in silicosis pathogenesis. The impairment of autophagy in AMs from Atg5^fl/fl^LysM^−^Cre^+^ silicosis model mice enhances NALP3 activity, aggravating silica-induced pulmonary fibrosis. These findings suggest that the silica-induced autophagy impairment is an important mechanism involved in the NALP3-mediated silicosis fibrosis. Intriguingly, in the Atg5^fl/fl^LysM^−^Cre^+^ silicosis mice model, the inhibition of autophagy elevates the release of IL-18 [12]. Furthermore, IL-18 neutralizing antibodies effectively reduce spontaneous disease in Atg7^−/−^ or Atg5^−/−^ mice [67]. In the future, the obstruction of the release of IL-18 mediated by autophagy may be involved in the silicosis treatment.

### 4.2. Exogenous Irritants-Mediated Autophagy Aggravates Silicosis Fibrosis

The characteristic of adsorption of silica may lead to the attachment of other “irritants”, which may have a joint toxic effect together with silica on pulmonary tissues. Our previous studies have demonstrated that many exogenous chemicals may exacerbate silicosis fibrosis through the regulation of autophagy. LPS is a characteristic component of the cell wall of Gram-negative bacteria. LPS has been detected in the air of coal mines in China, even in silicosis patients’ BALF. Moreover, LPS may induce accumulation of autophagosomes, aggravating the apoptosis of AMs in human silicosis. Notably, the expression of myeloid differentiation factor 88 (MyD88) and toll-like receptor adaptor molecule 1 (TLRAM1) elevate in AMs of humans treated with LPS, indicating that LPS-regulated autophagy may be dependent on TLR4-MyD88 or TLR4-TLRAM1 signaling pathway in human silicosis [50]. Moreover, the joint toxic effect of silica and LPS reveals the possibility that the components of silica are quite complex in the actual workplace.

Many workers’ exposure to silica involves a history of smoking, and smoking can enhance silica-induced AM apoptosis [32]. Furthermore, smoking aggravates the dysfunction of autophagic degradation in silicosis patients’ AMs, which may accelerate the progress of silicosis through increasing AM apoptosis [68]. As the main ingredient of smoke, nicotine reflects the levels of environmental tobacco smoke and second-hand smoke. We have also demonstrated that nicotine hampers the process of autophagic degradation in AMs of human silicosis. Blocked autophagic degradation caused by nicotine increases AM apoptosis in silicosis [69]. Therefore, silica exposure and smoking may enhance the damage to AMs and promote the development of silicosis. Providing a smoke-free workplace may be the potential protective measure for workers.

### 4.3. Autophagy Mediated by Natural Products Delays Silicosis Progression

In contrast to some toxic chemicals, several protective targets, therapy, or drugs have an anti-fibrosis function on silicosis (Table 2). Many studies indicate that exogenous administration of bone marrow-derived mesenchymal stem cells (BMSCs) may protect against various acute and chronic pulmonary diseases [70,71]. Further, in the silicosis rat model, the administration of BMSCs reduces AM autophagic activity, thereby effectively contributing to the fibrotic lung recovery [72].

Dioscin is the main component of traditional Chinese medicine Dioscorea. Recent studies have demonstrated that dioscin accelerates the process of autophagic degradation, further protecting AMs from mitochondria-dependent apoptosis. Simultaneously, the lack of autophagy function from Atg5 flox/flox Dppa3cre/+ mice reverses the alleviation of mitochondria dysfunction by dioscin. In terms of specific molecular mechanisms, dioscin activates beclin1 in response to the mitochondria dysfunction by silica inhalation, thereby increasing the expression of PINK1 and PARKIN, two executive proteins of mitophagy in AM of mice. AM mitophagy mediated by dioscin obliterates damaged mitochondria, further ameliorating silicosis fibrosis [73,74].

It has been recognized that silica activates macrophage autophagy activity. Moreover, two pro-fibrogenic factors, BBC3 and MCPIP1, may lead to macrophage apoptosis under silica circumstances through inducing autophagy activity. These findings support that the induction of autophagy activity may lead to the aggravation of silicosis fibrosis. However, the potential anti-fibrotic function by dioscin is mediated by the enhancement of macrophage autophagy activity, in contrast to the view that silica exerts a negative effect through the activation of macrophage autophagy. In these studies, the change of autophagy activity is often determined by the detection of the ratio of LC3-II/LC3-I. As mentioned before, the augmented expression of LC3-II may be caused by increased synthesis of autophagosomes or decreased turnover of autophagosomes (the binding of autophagosomes and lysosomes) [75]. Autophagy is a dynamic process, and the exploration between autophagy and silicosis fibrosis should be not limited to activated or inhibited autophagy alone. Therefore, more importance should be given to the obstructed/unobstructed process of autophagic degradation in the progression of silicosis.

Trehalose (Tre), a non-reducing disaccharide, has been regarded as a regulator of transcription factor EB (TFEB), which is also a regulator of autophagy-related gene transcription and lysosome biogenesis [76,77]. Knockout of TFEB aggravates silica-induced AM apoptosis by disruption of the autophagy-lysosomal system in the silicosis mice model. However, the nuclear transfer of TFEB activated by Tre relieves silica-induced lysosome damage and disorder of autophagic substrates degradation, reducing apoptosis in the mice-derived AM or AM of silicosis patients [11,78].

Atractylenolide III (ATL-III) is the active ingredient of the natural plant Atractylodes macrocephala Koidz. Similarly, ATL-III mitigates AM apoptosis by accelerating the process of autophagic degradation of human silicosis. Furthermore, inhibition of autophagy by ATL-III is regulated by the phosphorylation of the mammalian target of rapamycin (mTOR) [79]. ATL-III may be the first anti-silicosis fibrosis natural extract that inhibits mTOR-dependent autophagy. The improvement of the autophagic degradation induced by natural extracts may also be an effective intervention for silicosis fibrosis in the future. Interestingly, there may be multiple connections between mTOR and another autophagy regulator, Adenosine 5‘-monophosphate-activated protein kinase (AMPK). A study has shown that AMPK is inhibited through the phospholipase D-induced mTOR activation in tumor cells, thereby suppressing the autophagy flux [80]. These results may further reveal a potential intervention target for silicosis from the perspective of mTOR-dependent autophagy.

**Table 2:** Several potential autophagy-related targets in silica-induced pulmonary fibrosis.

Stimulus	Cell Type	Autophagy-Related Protein Level	Significance
Silica [49]	AMs from silicosis patients	Increased ratio of LC3II/I; Increased p62 level; Decreased LAMP2 level; Decreased TLR4 level	Silica leads to accumulation of autophagosomes and blockade of autophagic degradation in AMs of human silicosis
Pro-fibrotic Stimulus			
LPS [49]	AMs from silicosis patients	Increased ratio of LC3II/I; Increased p62 level; Increased MYD88 level; Increased TICAM1 level; Increased Beclin1 level	LPS aggravates silica-induced accumulation of autophagosomes and blockade of autophagic degradation in AMs of human silicosis
Smoking [67]	AMs from silicosis patients	Increased ratio of LC3II/I; Increased p62 level; Increased Beclin1 level	Smoking promotes silica-induced lack of autophagy function in AMs of human silicosis
Nicotine [68]	AMs from silicosis patients	Increased ratio of LC3II/I; Increased p62 level; Decreased LAMP2 level	Nicotine aggravates silica-induced lack of autophagy function in AMs of human silicosis
Potential anti-fibrotic targets			
MCPIP1-siRNA [60]	The human monocytic cell line U937 cells	Decreased ratio of LC3II/I; Decreased Beclin1 level; Decreased p53 level	MCPIP1-siRNA induces autophagy which is mediated by the p53 signaling pathway in macrophages exposed to silica
BBC-siRNA [57]	The human monocytic cell line U937 cells	Decreased ratio of LC3II/I; Decreased p62 level	BBC3-siRNA accelerates the process of autophagic degradation in macrophages exposed to silica
the exogenous administration of BMSCs [70]	AMs from rats	Decreased ratio of LC3II/I; Decreased Beclin1 level	Inhibition of autophagy is regulated by the administration of BMSCs in AMs of the silicosis rat model
Protective natural medicine			
Dioscin [72]	MH-S cell line	Increased ratio of LC3II/I; Decreased p62 level; Increased Beclin1 level; Increased PINK1 level; Increased PARKIN level	Dioscin-induced AM mitophagy protects against the mitochondria dysfunction by silica inhalation
Tre or TFEB Over-expression [11,77]	MH-S cell line or AMs from silicosis patients	Decreased ratio of LC3II/I; Decreased p62 level; Increased LAMP1 level	Nuclear transfer of TFEB activated by Tre improves disorder of autophagic substrates degradation in AM exposed to silica
ATL-III [78]	AMs from silicosis patients	Decreased ratio of LC3II/I; Decreased p62 level; Increased p-mTOR level	ATL-III protects the autophagy-lysosomal system by an mTOR-dependent pathway in AMs of human silicosis

## 5. Conclusions

It is determined that macrophage, especially AM autophagy, plays an important role in silicosis procession. Overactivated or overinhibited levels of autophagy activity are both harmful to the body. Only a proper level of autophagy activity maintains cell homeostasis balance. When autophagy regulators perform the protective function, we can see some induce macrophage autophagy activity, while others inhibit autophagy activity. However, the similarity of these substances or targets is that they all protect the process of macrophage substrate degradation in silicosis fibrosis. The blockade of autophagic degradation implies that a step of the autophagic process goes wrong, like an abnormal synthesis of autophagosomes, damage of lysosomes, or failed fusion of autophagolysosmes, etc. Overall, autophagy activity is judged largely according to the expression of LC3-II, but it cannot reflect the dynamic autophagy process. Therefore, a better description for future studies may be autophagy flux or autophagic degradation instead of autophagy activity.

In addition to the recommendation to better describe autophagy in silicosis, we also propose further exploring the detailed mechanism of macrophage autophagy in the development of silicosis. For example, how can we explain the dual effect of TLR4 targeting macrophage autophagy in the silicosis progression? Should the function of the p62-Keap1-Nrf2 axis and the interaction between p62 and TLR4 in the macrophage autophagy of silicosis be further explored? Meanwhile, is the knockdown of BBC3 or NALP3 expected to be an intervention target for silicosis? From the perspective of workplace exposure to silica, our previous study shows that smoking may enhance the harm to AMs and silicosis fibrosis. Considering the characteristic of adsorption of silica, LPS may perform a combined toxic effect with silica. Therefore, the complex components of silica and substances that exist in the air of the work environment deserve further discussion. Additionally, based on the current scientific data, many natural product ingredients have been proved to delay the development of silicosis fibrosis through the regulation of autophagy. In the future, we recommend further studies on potential targets or drugs for intervention in silicosis.

## Figures and Tables

**Table 1:** Comparison of different silica-induced mice or rat silicosis models.

Methods	Route of Administration	Advantages	Disadvantages
Exposed or non-exposed intratracheal instillation [9,14]	Instillation of SiO_2_ suspension into the trachea	One-time instillation; Simple operation; Low cost	Cannot reflect the mechanism of actual human silicosis; The exposed way may increase animal infection
Dynamic or static inhalation [15,16]	Inhalation of SiO_2_ aerosol	Better for simulating the pathologic process of actual human silicosis	Long modeling time; Expensive equipment
